# Healing of Periapical Lesions After Surgical Endodontic Retreatment: A Systematic Review

**DOI:** 10.7759/cureus.6916

**Published:** 2020-02-07

**Authors:** Faisal Alghamdi, Abdulrahaman J Alhaddad, Samar Abuzinadah

**Affiliations:** 1 Oral Biology, King Abdulaziz University, Jeddah, SAU; 2 Oral and Maxillofacial Rehabilitation, King Abdulaziz University, Jeddah, SAU; 3 Conservative Dentistry, King Abdulaziz University, Jeddah, SAU

**Keywords:** healing, endodontic surgery, apical surgery, surgical retreatment, endodontic treatment, periapical lesion

## Abstract

Background: Surgical root canal retreatment is required when peri-radicular pathosis associated with endodontically treated teeth cannot be treated by non-surgical root canal therapy (retreatment), or when retreatment was ineffective, not feasible or contraindicated. Endodontic failures maybe happen when irritants remain within the confines of the root canal, or when an extra-radicular infection cannot be eradicated by orthograde root canal treatment. Following enhanced microsurgical techniques in the last years, the success rates of surgical root canal retreatment have improved considerably.

Objective: The aim of this systematic review is to gather updated data in regard to the surgical root canal (retrograde) retreatment to heal the periapical lesions.

Materials and methods: The electronic databases PubMed and Google Scholar were searched in this review using specific inclusion and exclusion criteria. The search was performed in June 2019 and updated in November 2019. Among 3900 studies, 10 studies satisfied the eligibility criteria and were included in the review to be analyzed.

Results: The 10 studies showed the importance of surgical root canal retreatment as a treatment option in removing infections within the root canal system and its efficiency in periapical tissue healing. These studies investigated different aspects of healing of periapical lesion after surgical (retrograde) retreatment including success rates, follow-up duration, and updated studies in surgical (retrograde) retreatment.

Conclusions: Surgical root canal (retrograde) retreatment demonstrates its efficiency in reducing the period needed for healing of the periapical lesions in short-term follow-up compared to conventional orthograde retreatment.

## Introduction and background

Periapical lesions are one of the common pathological conditions affecting periradicular tissues [1]. The microbial invasion and subsequent infection of the canal systems of a root play a decisive role in the initiation and progression of periapical lesions [2]. Periapical lesions are mostly classified as radicular cysts, dental granulomas or abscesses [3,4]. Among all periapical lesions, the incidence of cysts varies from 6% to 55% [5]. Also, the occurrence of granulomas spans from 9.3% to 87.1%, and of abscesses from 28.7% to 70.07% [6]. According to clinical evidence, lesions that are larger in size, are most likely radicular cysts. Still, some of these large lesions may appear to be granulomas [7].

The preliminary purpose of all endodontic procedures, especially cleaning and shaping, is to eliminate necrotic tissue and infective bacteria [8]. Large periapical lesions are of inflammatory origin as well as apical true cysts and should be treated initially with a nonsurgical approach [9]. When intra- or extra-radicular infections are persistent, and periapical pathology fails to resolve after nonsurgical endodontic management protocols, only then a surgical option should be considered [10]. Broad cross-sectional studies from various countries have stated that the prevalence of apical periodontitis and other post-treatment periradicular diseases can transcend 30% of all root-filled teeth population [11-14]. These facts recommend a significant requirement for the treatment of this condition [11-14]. Microsurgical endodontic treatment is better than conventional endodontic treatment and has high success rates [15].

There are several studies that were conducted to discuss the healing of periapical lesion after nonsurgical (orthograde) retreatment or surgical root canal treatment. However, few studies have investigated the healing of periapical lesion after surgical (retrograde) retreatment. Consequently, the aim of this review was to collect all updated and available studies including imperative information concerning the surgical root canal (retrograde) retreatment to heal periapical lesions.

## Review

Material and methods

This review has been compiled according to the Preferred Reporting Items for Systematic Reviews and Meta-analyses (PRISMA) guidelines.

Research Question

The following was the research question for the systematic review: “The best endodontic treatment option for the healing of periapical lesions: is it surgical retrograde retreatment or conventional orthograde retreatment?”.

Literature Search

With respect to the question of the study, we searched the literature and identified relevant studies. The literature search was formulated in June 2019 and then updated in November 2019. A web search was done through PubMed (2009-2019) and Google Scholar (2009-2019) with MesH terms and/or in various combinations (“healing”, “periapical lesion”, “surgical root canal retreatment OR surgical endodontic retreatment”, “endodontic microsurgery retreatment”).

Relevant articles had been read and assessed by the introduction of the close meaning ideas by the study reviewers. Full articles were obtained for most of the titles and abstracts that met the inclusion criteria, the full text was accessed. From each included article, study design, interventions, and findings were extracted. Articles used were categorized into two main groups (free and restricted). Free ones have been downloaded directly by the URLs generated from the database. The restricted group has been downloaded by the institutional access of the King Abdulaziz University (KAU) library. Even though some articles did not match the main idea, they have been reviewed again & decided to be either relevant or irrelevant.

Inclusion Criteria

1. Native research released in the English language.

2. Time framed articles released within 10 years (2009 - 2019).

3. Studies carried out on human subjects only.

Exclusion Criteria

1. Articles that described healing of periapical lesion with management techniques excluding the surgical root canal retreatment.

2. Articles that discussed healing of periapical lesion after surgical root canal retreatment by percentages and samples taken from animals.

3. Review articles.

Critical Appraisal

Eligible studies were independently analyzed by all reviewers according to the eligibility criteria as well as PRISMA guidelines. Any disagreement between the reviewers was resolved using discussion.

Data Extraction and Presentation

**Figure 1:**
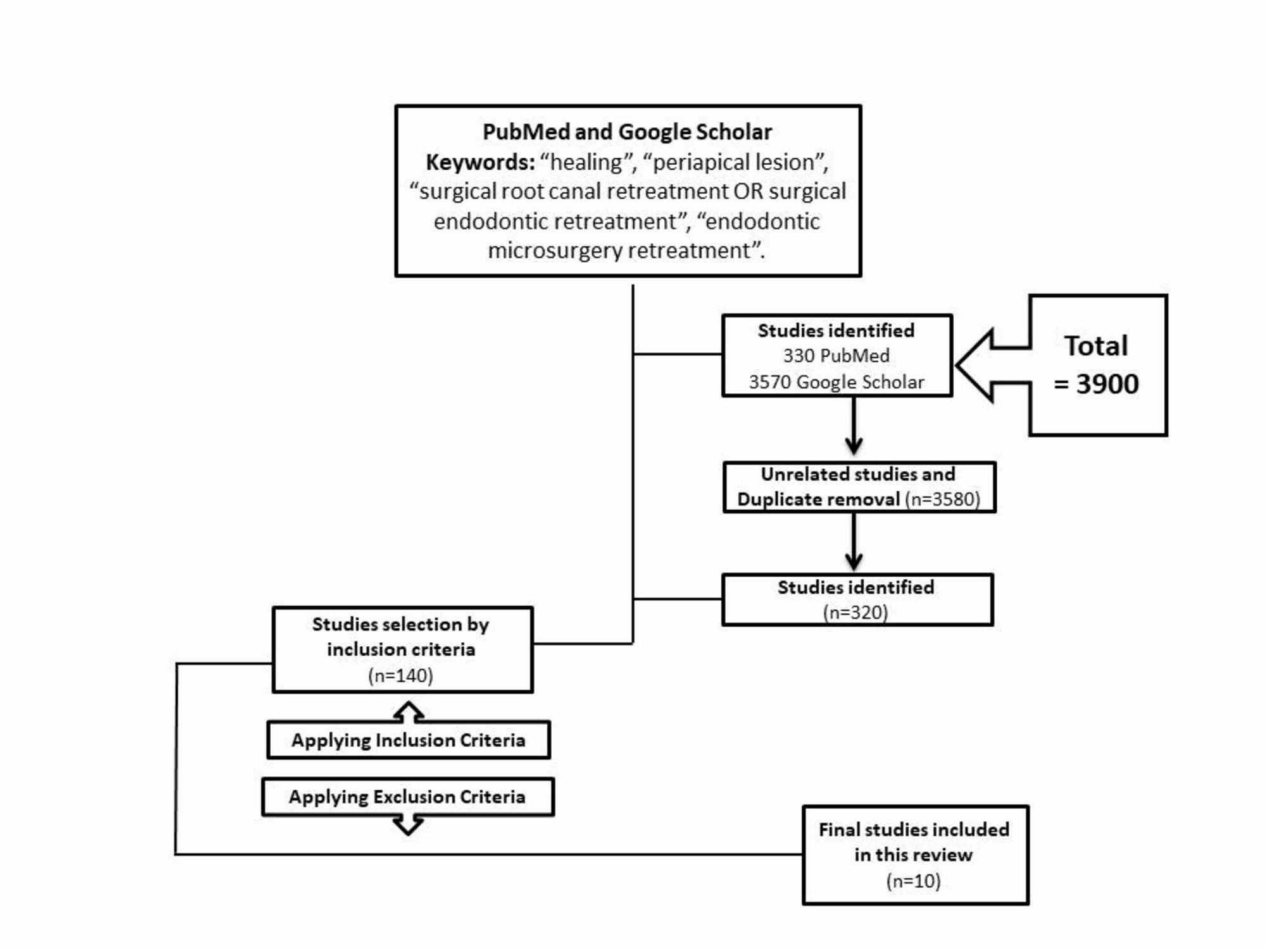
Flow Chart of the Search Strategy Used in this Systematic Review

The search strategy using the keywords and MeSH of the databases like PUBMED and Google Scholar yielded a total of 3,900 studies, of which 3,580 were either unrelated or duplicate topics. Among the potential 140 studies, the eligibility criteria were applied and ten studies were included in this systematic review. The summary of the search flow chart for this systematic review has been depicted in (Figure 1).

Results

The search culminated in 10 studies that fulfilled both the inclusion and exclusion criteria and which were conducted in the last 10 years ago. These studies investigated the healing of periapical lesion after surgical root canal (retrograde) retreatment including success rates, follow-up duration, and updated studies in surgical root canal (retrograde) retreatment. The studies included in this systematic review were one randomized controlled trial study, two prospective studies, one retrospective study, and six case reports [16-19,20-25]. The systematic review included ten studies with a total sample of 376 subjects that were treated from primary care centers and also universities outpatient departments of dental schools and hospitals. In all of the studies, the procedures were performed on systemically healthy persons. In regard to the duration of follow-up performed, one study ranged from 1 to 3 year recalls, four studies were performed with 1-year recall and different studies up to “2 years recalls”, “5 year recalls”, “6 year recalls”, and “10 year recalls” [16-25]. In regard to the surgical technique performed, the placement of root-end filling material was made in four studies, and in the other six studies, root-end filling material was not placed [16-25]. In regard to the effect on the healing of the periapical lesions, all the studies showed a high significant success rate of complete healed or remained healed of the periapical lesion after surgical retrograde retreatment [16-25]. In regard to the most success rate of endodontic surgery, two studies found that microsurgical techniques had a high success rate in healing the periapical lesions compared to conventional orthograde treatment [23,24]. All included studies were summarized in Table 1. A summary of all current systematic and meta‑analysis reviews are summarized in Table 2.

**Table 1:** Summary of All Included Studies in the Systematic Review

Authors/Study Design	Year	Number of Subjects	Healing (Yes / No)	Duration of Follow-up	Main Results	Main Conclusion
Kruse C et al. [16], Denmark, (Randomized Controlled Trial study)	2016	(n= 44)	(Yes)	“1 year” + “A 6-year Follow-up”.	In the recall visit after 6 years, 90% of the teeth in the GP group that were scored as effectively recuperated 12-months postoperatively stayed asymptomatic. In the MTA group, 80% of the teeth studied as adequately repaired following 12-months stayed asymptomatic.	Revelations demonstrate that a 12-months follow-up may not be adequate in evaluating the long-term result of surgical endodontic retreatment. With an extended follow-up, different determinants not clearly associated with the endodontic treatment might be appropriate for an effective result.
Shinbori N et al. [19], USA, (Retrospective study)	2015	(n= 94)	(Yes)	“Ranged from 1 to 3 years”.	All-inclusive the success rate was 92.0% after the endodontic microsurgery.	The use of ES-BCRR as a root canal filling material resulted in a favorable repair rate of 92.0% in endodontic microsurgery at least 12-months recall investigation.
Machado R et al. [20], Brazil, (Case Report)	2014	Two Case Report	(Yes)	Case No.1: “9 months” + “1-year follow-up”. Case No. 2: “Follow-up examinations every 3 months for a year”.	In the current cases, the outcome of the microsurgical techniques in lesion regression and tooth survival 1 year post-treatment.	It fortifies the statute that combining finding out about the biologic aspects of endodontics with surgical endodontic treatment using a modern technique is an elective foreseen treatment.
PFE Bernabe et al. [21], Brazil, (Case Report)	2013	Case Report	(Yes)	“A 5-year Follow-up”	At the 5-year follow-up, there were no clinical signs or symptoms related to the lesion, and the radiographic examination indicated a growing resolution of the radiolucency.	It might be presumed that MTA presents favorable qualities in unfavorable conditions and could be used in combination with GTR in cases including root reconstruction.
Song M et al. [17], South Korea, (Prospective Follow-up study)	2012	(n= 172)	(Yes)	“Followed up every 6 months for 24 months and every year up to 10 years”.	Of the 104 followed-up cases, the successful group had 97 cases, 91 of which had complete healing and 6 had incomplete healing. The general maintained success rate was 93.3%.	In a previous 5-year study, 93.3% of endodontic microsurgery cases that were considered successfully treated stayed the same after more than 6 years.
Brito-Junior M et al. [22], Brazil, (Case Report)	2012	Case Report	(Yes)	“6 months” + “1 year” + “2 year follows-up”	A radiograph was taken following a half year exhibited progressed periapical healing in the current case. Be that as it may, a complete repair was noted at only one year postsurgery, and complete periapical repair at the two-year follow-up.	Based on these clinical and radiographic aspects, the apical surgical intervention proved to be a successful treatment to overcome the failure of the conservative approach used in this case.
Kahler B. [23], Australia, (Case Report)	2011	Case Report	(Yes)	“1-year follow-up”	Healing was evident at a 1-year review appointment.	The overall healing of periapical lesions demonstrated superior results when treated with microsurgery contrasted with conventional techniques to endodontic surgery. Success rates have appeared to be comparable with traditional orthograde treatment.
Song M et al. [18], South Korea, (Prospective Clinical study)	2011	(n= 54)	(Yes)	“Every 6 months for 2 succeeding and Every year”	42 cases were recalled, 39 of which were included in the success category, giving an overall success rate of 92.9%.	The use of microsurgical techniques and biocompatible materials such as MTA and Super-EBA outcomed in a high clinical success rate, even in endodontic re-surgery.
Kahler B [24], Australia, (Case Report)	2010	Five Case Reports	(Yes)	“1 year follow-up”	Healing was obvious at 12-month recall. Microsurgical techniques have significantly improved the results for healing of periapical lesions when contrasted with traditional approaches to endodontic surgery.	Success rates were found to be comparable with conventional orthograde treatment.
Karabucak B et al. [25] USA, (Case Report)	2009	Two Case Reports	(Yes)	Case No.1: “One-year and 2-year recalls”. Case No.2: “At a 1-year recall”.	Case No.1: Radiographic assessments showed complete periapical bone healing when microsurgery was utilized. Case No.2: Normal results were found for clinical and radiographic examinations.	These cases show successful surgical treatment of combined lesions.

**Table 2:** Summary of All Current Systematic and Meta‑analysis Reviews in the Literature

Authors/Study Design	Year	Number of studies using	Method summary	Main Conclusions
Kang M, et al. [26], South Korea (Systematic Review)	2015	18 studies	The systematic review summaries and presents Clinical studies performed from January 1970 to June 2012. Using 4 different databases (PubMed, Embase, Medline, and The Cochrane Library), and analysis of the papers published during this period took place based on previously established criteria, by means of the methodology of a systematic review.	Endodontic microsurgery was confirmed as a reliable treatment option with favorable initial healing and a predictable result.
Del Fabbro M, et al. [27], Brazil (Review)	2007	3 controlled clinical trials	The systematic review summaries and presents clinical studies until the 3rd of April 2007, using 3 different databases searched: (The Cochrane Library, MEDLINE and EMBASE) an electronic search was done.	The finding that healing rates could be higher for cases treated surgically as compared to that treated non-surgically, at least in the short term.
Torabinejad M, et al. [28], USA (Systematic Review)	2009	34 studies	A systematic review summary and presents original articles in the MEDLINE, PubMed, and Cochrane databases, The publication date ranged from 1998-2008 for nonsurgical retreatment literature and 1970–2008 for the endodontic surgery literature; an electronic search was done.	Endodontic surgery offers more favorable initial success, but nonsurgical retreatment offers a more favorable long-term outcome.

Discussion

The systematic review presents a comprehensive compilation of evidence taken from ten articles which included original studies. The sample size was up to 376 subjects seeking endodontic retreatment by the use of surgical retrograde retreatment. All included studies confirmed faster treatment time by surgical root canal (retrograde) retreatment (Table 1). The recently published systematic reviews by Kang M et al. in 2015 illustrated “The endodontic microsurgery and nonsurgical retreatment have stable results showing the overcome of pooled success rates at about 92% and around 80%, respectively” (Table 2) [26]. When the data were reviewed and analyzed in the follow-up periods, they found the microsurgery group had a significantly superior success rate than the retreatment group in the short-term follow-up, while no significant difference was improved in the long-term follow-up [26].

Also, Del Fabbro et al. in 2007 and Torabinejad et al. in 2009 have compared the success rates of non-surgical orthograde and surgical retrograde endodontic retreatment [27,28]. They found the surgically treated cases seem to indicate a higher success rate after one year. However, after 2-4 years relative success rates show equivalent or reversed [27,28]. This clearly shows agreement in the conclusions reached by these systematic/meta‑analysis reviews in regards to the healing of periapical lesions by surgical retrograde retreatment compared to the conventional orthograde retreatment especially after one year of procedure (short-term follow-up). When examining our included studies individually, 10 studies favored the use of surgical root canal (retrograde) retreatment, whereas most of the studies concluded that there was a high significant success rate of complete healed or remained healed of periapical lesions after the (retrograde) retreatment in short-term follow-up, but there are some studies showed significant healing of periapical lesions after different long-term follow-ups [16-25]. In addition, two studies found that microsurgical techniques have a high success rate in healing of the periapical lesions compared to conventional orthograde treatment [23,24]. Our results clearly reveal the controversy in the literature; however, there is, indeed, a strong trend toward supporting the microsurgical retreatment. Hence, more studies are needed to formulate the proper guidelines and parameters of how and when surgical retrograde retreatment can be used and considered as an accurate and reliable treatment option to heal the periapical lesions.

## Conclusions

Surgical root canal (retrograde) retreatment is defined as an important invasive procedure that permits fast treatment options minus the necessity of the extensive traditional method. Surgical retrograde retreatment demonstrates its efficiency in reducing the period needed for healing of the periapical lesions and suggests benefits that will result in better recognition among patients seeking faster results in short-term follow-up, but on the long-term follow-up showed not significant difference for healing of periapical lesions compared to conventional orthograde retreatment. However, more clinical trials are encouraged to inspect the results of surgical retrograde retreatment on the healing of periapical lesions.
